# Determinants of disease activity change over time in Enthesitis related arthritis: effect of structured outcome monitoring and clinical decision support

**DOI:** 10.1186/s12969-020-00472-3

**Published:** 2020-10-15

**Authors:** Francesca Tirelli, Rui Xiao, Timothy G. Brandon, Jon M. Burnham, Joyce C. Chang, Pamela F. Weiss

**Affiliations:** 1 Department of Pediatrics, Rheumatology Unit, Anna Meyer Children’s Hospital and Department of Women’s and Children’s Health, University of Padova, Via Giustiniani 2, 35128 Padova, Italy; 2 Department of Biostatistics, Epidemiology and Informatics, Perelman School of Medicine at the University of Pennsylvania, 423 Guardian Drive, Philadelphia, PA 19104 USA; 3 Department of Pediatrics, Division of Rheumatology at the Children’s Hospital of Philadelphia, Wood Bldg 1st foor, 3401 Civic Center Blvd, Philadelphia, PA 19104 USA; 4 Department of Pediatrics, Division of Rheumatology at the Children’s Hospital of Philadelphia, Perelman School of Medicine at the University of Pennsylvania, Wood Bldg 1st foor, 3401 Civic Center Blvd, Philadelphia, PA 19104 USA; 5 Department of Pediatrics, Division of Rheumatology at the Children’s Hospital of Philadelphia and Center for Clinical Epidemiology and Biostatistics, Perelman School of Medicine at the University of Pennsylvania, Wood Bldg 1st foor, 3401 Civic Center Blvd, Philadelphia, PA 19104 USA; 6 The Children’s Hospital of Philadelphia, Roberts Center for Pediatric Research, 2716 South Street, Room 11121, Philadelphia, PA 19104 USA

**Keywords:** Ankylosing spondyloarthritis, juvenile idiopathic arthritis, Biologic therapy, Decision support systems, clinical

## Abstract

**Background:**

We aimed to test if standardized point-of-care outcome monitoring and clinical decision support (CDS), as compared to standard care, improves disease activity and patient-reported pain in children with enthesitis-related arthritis (ERA).

**Methods:**

This was a retrospective cohort study of outcomes of children with ERA after phased implementation of I) standardized outcome monitoring with CDS for polyarticular JIA, and II) CDS for ERA, compared to a pre-intervention group of historical controls. We used multivariable mixed-effects models for repeated measures to test whether implementation phase or other disease characteristics were associated with change over time in disease activity, as measured by the clinical juvenile arthritis disease activity score (cJADAS), and pain.

**Results:**

One hundred fifty-two ERA patients (41% incident cases) were included with a median age of 14.9 years. Implementation of standardized outcome monitoring or ERA-specific CDS did not result in significant differences in cJADAS or pain over time compared to the pre-intervention cohort. Higher cJADAS at the index visit, pain and more tender entheses were significantly associated with higher cJADAS scores over time (all *p* < 0.01), while biologic use was associated with lower cJADAS (*p* = 0.02). Regardless of intervention period, incident ERA cases had a greater rate of cJADAS improvement over time compared to prevalent cases (*p* < 0.01), but pain persisted over time among both incident and prevalent cases.

**Conclusions:**

There was no significant effect of point-of-care outcome monitoring or CDS interventions on disease activity or pain over time in children with ERA in this single center study. Future efforts to improve disease outcomes using standardized outcome monitoring and CDS will need to consider the importance of addressing pain as a target in addition to spondyloarthritis-specific disease activity metrics.

## Background

Juvenile idiopathic arthritis (JIA) is the most common pediatric rheumatologic disease, and is classified into seven different categories by the International League of Associations for Rheumatology (ILAR). The category known as enthesitis-related arthritis (ERA) accounts for 10–20% of JIA and is characterized by the association of arthritis, enthesitis, involvement of sacroiliac joints and axial skeleton and strong correlation with the presence of human leukocyte antigen (HLA) B-27 [1, 2].

When compared to the other categories of JIA, patients with ERA tend to have poorer outcomes, including higher disease activity, decreased likelihood of achieving prolonged remission, increased pain and lower health status [3–5]. Therapeutic strategies for ERA are largely based on knowledge from adult spondyloarthritis, and variability in treatment approach has been described among pediatric rheumatologists [4, 6].

Treat-to-target (TTT) is a therapeutic approach that consists of tight disease activity monitoring with standardized measures and adapting treatment strategies accordingly to reach a well-defined treatment target. In the last decade, this approach has been demonstrated to improve disease outcomes for rheumatologic diseases and has therefore been included in treatment recommendations for rheumatoid arthritis and ankylosing spondylitis [7–9]. Recently, an international task force recommended implementation of TTT strategies for the treatment of JIA [10]. Moreover, protocolized treatment strategies may be even more effective in inducing disease control [11]. The use of computer-based clinical decision support (CDS) systems is one strategy to standardize treatment decisions. In the CAMERA trial [12], a CDS system was used to adjust methotrexate dosages in patients with early rheumatoid arthritis in a TTT intervention; this strategy had greater clinical efficacy compared to standard of care. A recent study conducted at our center demonstrated that the implementation of a TTT strategy with standardized outcome monitoring augmented by clinical decision support treatment algorithms determined a significant improvement in disease activity and patient reported outcomes for children with polyarticular JIA [13].

The objectives of this study were to test if implementation of standardized point-of-care disease activity monitoring and CDS for polyarticular JIA, as compared to standard care, improved disease activity and patient-reported pain in children with prevalent and incident ERA. We also tested if implementation of CDS tailored for children with ERA further augmented the impact of the initial intervention.

## Methods

**Fig. 1:**
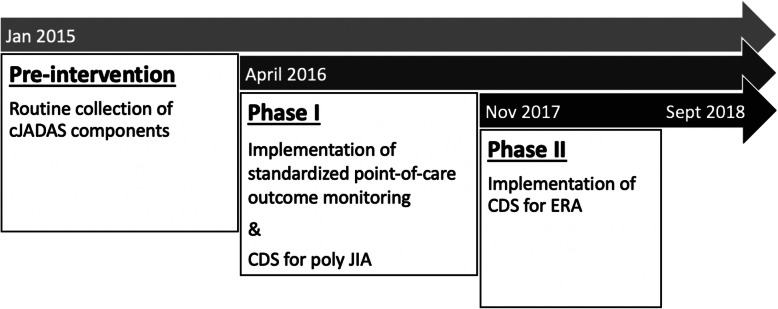
Study cohorts defined by phased implementation of standardized outcome monitoring and clinical decision support. Routine collection of cJADAS components started in January 2015. The CDS algorithm for polyarticular JIA was introduced in April 2016, shortly after implementation of standardized point-of-care outcome monitoring. The CDS algorithm for ERA was implemented in November 2017

With the aim of improving disease outcomes among children with JIA, a quality improvement initiative was launched in the Division of Rheumatology of CHOP in 2016. The first phase of the intervention, detailed elsewhere [13], included standardized disease activity measurement with the clinical juvenile arthritis disease activity score cJADAS [14], point-of-care disease outcome assessment, and integration of a CDS tool for polyarticular JIA to reduce treatment variability. The second phase of the intervention included implementation of a CDS tool for the ERA subtype of JIA (Fig. 1).

### Standardized disease outcome assessment

In February 2016, we first launched an outcome assessment method for all JIA patients using Research Electronic Data Capture (REDCap) [15] survey tools hosted at the Children’s Hospital of Philadelphia to collect patient/parent and physician components of the cJADAS, Patient-Reported Outcomes Measurement Information System (PROMIS) physical function pediatric and parent proxy short forms [16, 17], and pain scores (0–10, visual analog scale) (Fig. 1). Patient-reported outcomes (PROs) were provided electronically by caregivers of patients under eight years old or by patients eight years of age and older prior to the encounter; active joint count and physician global assessment were collected from the treating physician after the clinical evaluation. Caregiver/patient responses and physician global assessments in REDCap were made accessible via a direct link from the patient’s electronic health record (EHR), allowing a point-of-care review of the PROs and cJADAS score by the treating physician. At the end of the encounter, providers performed a target attestation in the EHR to acknowledge that they had reviewed the patient’s scores, and to indicate whether the goal treatment target had been achieved using one of three options to characterize disease activity: “not active and at target”, “active and not at target”, or “active but at target”. The treatment target for each patient was determined by the treating providers such that patients with some degree of active disease could still be considered “at target” if the pre-specified treatment goal was to achieve low disease activity rather than inactive disease. Patient history and physical examination could also factor in to the physician’s assessment of whether the patient was at target.

### Clinical decision support

**Fig. 2:**
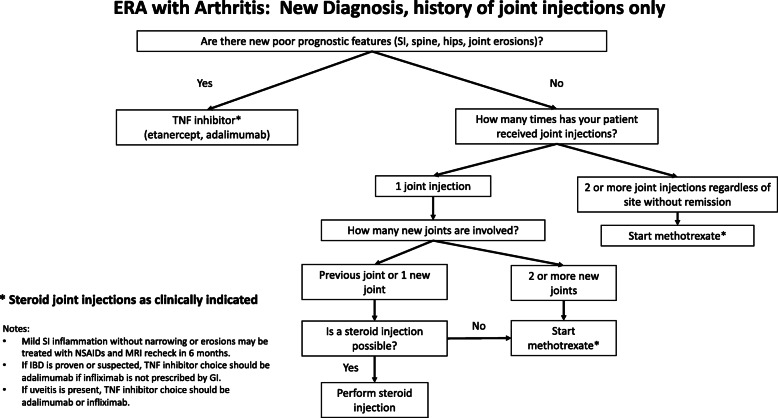
Example of an ERA CDS algorithm for child with incident diagnosis and no history of systemic therapy

The standardized point-of-care disease activity outcome assessment was augmented by the implementation of CDS algorithms with the aim of reducing the variability of JIA treatment by standardizing medication selection, dosing, and treatment duration. The Phase I algorithms implemented in April 2016 were initially developed for patients with polyarticular JIA and had varying pathways for different stages of disease based on current treatment consensus and adapted for use in the local context [13]. The Phase II CDS algorithms for ERA were implemented in November 2017. CDS for ERA included algorithms based on the disease status (new diagnosis, flare, remission) with different treatment suggestions for patients with active disease (new diagnosis or disease flare) dependent on the type of joint involvement (axial vs. peripheral), severity of symptoms, presence of poor prognostic factors, previous medications (in the case of flares). Algorithms for patients in remission included suggestions regarding treatment duration and medication tapering. Indications for medication selection and optimal dosage for each drug considered in the algorithms were provided. Branching logic in the physician component of the REDCap tool allowed providers to refer to the CDS algorithms at the point of care. The algorithm for an incident case with no prior systemic therapy is shown in Fig. 2.

### Study population

In this retrospective study, we included children with prevalent (disease duration ≥6 months) and incident (disease duration < 6 months) ERA who were being followed in a CHOP rheumatology clinic before and/or after the implementation of the CDS initiative. ILAR category was confirmed by manual chart review for all subjects.

We defined three intervention cohorts based on the implementation stages of the point-of-care disease activity outcome assessment and CDS interventions – pre-intervention, Phase I, and Phase II cohorts (Fig. 1). The pre-intervention cohort included patients with ≥2 visits at least one month apart between January and December of 2015, during which components of the cJADAS [14] and pain scores were routinely collected in the EHR, but there was no standardized assessment of cJADAS or a CDS tool. The Phase I cohort included patients with ≥2 visits at least one month apart between April 2016 (when CDS for polyarticular JIA was first implemented in addition to standardized outcome monitoring) and September 2018. In order to assess the impact of the ERA-specific CDS, we also defined a Phase II cohort, limited to patients with ≥2 visits after implementation of the ERA-specific CDS in November 2017. The index visits were defined as the first visit in 2015 for the pre-intervention cohort, the first visit after the polyarticular JIA CDS implementation date for the Phase I cohort, and the first visit after the ERA CDS implementation date for the Phase II cohort.

### Data source

Data for the pre-intervention cohort were abstracted from the EHR. Data for the Phase I and II cohorts were abstracted from the Qlikview Platform, an automated data visualization tool that integrates the REDCap survey data for each JIA visit into the EHR.

### Study measures

Cases were categorized as having an incident diagnosis (≤ 6 months since initial JIA diagnosis) or prevalent diagnosis (> 6 months since diagnosis) at the time of the index visit. Time in months following the index visit was the primary exposure. The primary outcome was disease activity, as defined by the cJADAS. The 10-joint cJADAS (cJADAS10) is a three-element disease activity score which has been validated for non-systemic JIA, including ERA [14]. The score is computed by measuring active joint count (maximum 10 joints), physician global assessment (0–10), and parent/patient global assessment (0–10). Scores range from 0 to 30 with higher scores indicative of higher disease activity. Patients who score ≤ 1 are considered to have inactive disease with thresholds for low, moderate, and high disease activity differing dependent on if the patient has oligo- or polyarticular disease. Low, moderate, and high disease activity are defined as ≤1.5, > 1.5 to ≤4, and > 4 for oligoarticular disease, and ≤ 2.5, > 2.5 to ≤8.5, and > 8.5 for polyarticular disease [14].

Covariates included the following time-invariant characteristics: demographics (age in years, sex, race, ethnicity, commercial versus public insurance), HLA B27 status, oligo- versus polyarticular course, and history of sacroiliitis (confirmed by MRI); and time-varying disease features measured at each visit: pain score, tender entheses count, active uveitis, biologic use, disease modifying antirheumatic drug [DMARD] use, intraarticular injection. Standard tender entheses evaluation included 14 entheses: common flexor tendon insertion on medial elbow epicondyle (left/right (L/R)), common extensor tendon insertion on lateral elbow epicondyle (L/R), greater trochanter (L/R), quadriceps insertion on the superior pole of patella (L/R), patellar ligament insertion into inferior pole of patella (L/R), Achilles tendon insertion on the calcaneus (L/R), plantar fascia insertion on the calcaneus (L/R).

### Statistical analysis

Descriptive statistics were used to summarize baseline demographic factors and disease activity. We used linear mixed-effects models to test the association between intervention cohort and cJADAS scores over time, as well as the association between intervention and pain scores. This model accounts for within-subject correlation due to repeated measures. Separate models were used to compare the pre-intervention cohort to those in each implementation phase. For all models we adjusted for time and baseline cJADAS scores, and employed a first-order autoregressive (AR1) covariance structure which assumes the correlation between two adjacent measures declines exponentially as the time between measures increases. Covariates were tested in ‘univariate’ mixed-effects regression models with time included, and those with *p* values < 0.2 were considered in multivariable models. The stepwise forward selection algorithm was used to determine the final multivariable models. To determine whether the rate of change in disease activity over time differed by intervention period, we tested for interactions between time and cohort (pre/post intervention) in the regression models. We also tested for interactions between time and disease duration (incident vs prevalent disease) and interactions between entheses count and pain. In a secondary analysis, we used separate linear mixed-effects models to evaluate individual components of the cJADAS over time.

In a sensitivity analysis, we restricted comparison of the pre-intervention CDS and Phase II cohorts to include only visits at which target attestation was completed. All statistical analyses were conducted using Stata, version 15 (Stata Corporation, College Station, TX).

## Results

### Baseline characteristics

Demographics and baseline disease characteristics are shown in Table 1. 152 ERA patients met inclusion criteria, with a median age of 14.9 years (interquartile range [IQR]: 12.2, 17.4). The pre-intervenion cohort included 54 patients, 28% of which were incident cases. The median number of visits was 2 (IQR: 2, 3) over a median of 6 months (IQR: 4, 9). Nine children (22 visits; 13.6%) were excluded due to missing one or more cJADAS components. The Phase I cohort included 98 patients, with 32% incident cases; median number of visits was 4 (IQR: 2, 5) over a median of 21 months (IQR: 11, 24). All visits had complete cJADAS components. Providers completed target attestation for 67.4% of post-poly CDS visits. The proportion of incident and prevalent cases as well as the demographics, medication use, and ERA criteria fulfilled were similar in the pre-intervention and Phase I cohorts. Baseline reported pain was lower in the pre-intervention cohort, while a greater proportion of the Phase I group was publicly insured.

**Table 1:** Baseline demographic and clinical characteristics before and after implementation of standardized outcome monitoring and CDS for polyarticular JIA

	All *N* = 152	Pre-intervention *N* = 54	Phase I *N* = 98	*p*-value
Incident diagnosis, n (%)	46 (30)	15 (28)	31 (32)	0.62
Age, median (IQR)	14.9 (12.2–17.4)	15.5 (12.1–16.8)	14.8 (12.2–17.5)	0.79
Male, n (%)	98 (64)	35 (65)	63 (64)	0.95
Race (Non-white) *n* (%)	23 (15)	7 (13)	16 (16)	0.58
Hispanic Ethnicity, *n* (%)	9 (5)	4 (7)	5 (5)	0.56
Public insurance, *n* (%)	67 (44)	17 (31)	50 (51)	0.02
ILAR Criteria for ERA				
•Presence of arthritis, *n* (%)	131 (86)	47 (87)	84 (86)	0.82
•Presence of enthesitis, *n* (%)	129 (85)	48 (89)	81 (83)	0.31
•Arthritis in a male > 6 year, *n* (%)	68 (45)	26 (48)	42,843)	0.53
•SIJ tenderness, *n* (%)	64 (42)	24 (44)	40 (41)	0.67
•Acute anterior uveitis, *n* (%)	14 (9)	4 (7)	10 (10)	0.57
•Family history of HLA-B27 related disease, *n* (%)	12 (8)	5 (9)	7 (7)	0.64
Baseline pain^a^, median (IQR)	1.9 (0.1–4.9)	1.0 (0.0–3.0)	2.1 (0.4–5.3)	0.02
Baseline cJADAS^b^, median (IQR)	3.0 (0.9–8.0)	3.0 (1.0–6.0)	4.5 (0.6–8.3)	0.09
•Active Joint Count^c^	0.0 (0.0–1.0)	0.0 (0.0–1.0)	0.0 (0.0–1.0)	0.25
•Physician’s global assessment^d^	1.0 (0.0–3.0)	0.0 (0.0–2.0)	1.0 (0.0–3.0)	0.07
•Patient/parent global assessment^e^	1.8 (0.3–3.9)	1.5 (0.0–3.0)	1.9 (0.4–4.1)	0.27
Baseline tender entheses count^f^, median (IQR)	0.0 (0.0–2.0)	0.0 (0.0–2.0)	0.0 (0.0–1.0)	0.11
HLA – B27 positive^g^, *n* (%)	62 (42)	19 (36)	43 (46)	0.24
Polyarticular disease, *n* (%)	42 (28)	16 (31)	26 (27)	0.58
Sacroilitis confirmed by MRI (ever), *n* (%)	42 (28)	15 (28)	27 (28)	0.97
Biologic use (ever)	101 (66)	35 (65)	66 (67)	0.75
DMARD use (ever)	58 (38)	20 (37)	38 (39)	0.83

^**a**^Pain visual analog scale score (range 0,10); ^b^cJADAS = three-variable clinical Juvenile Arthritis Disease Activity Score (range 0,30); ^c^Active joint count (range 0,10); ^d^Physician global assessment of disease activity visual analog scale (range 0,10); ^e^Patient/parent global assessment of disease activity visual analog scale (range 0,10); ^f^ (range 0,14) ^g^B27 status missing for 4 subjects

*Abbreviations*: *CDS* Clinical Decision Support, *IQR* interquartile range, *ILAR* International League of Associations for Rheumatology, *ERA* Enthesitis Related Arthritis, *SIJ* Sacroiliac Joints, HLA Human Leukocyte Antigen, *MRI* Magnetic Resonance Imaging, DMARD Disease modifying Anti Rheumatic Drug

### Impact of clinical characteristics and treat-to-target intervention on disease activity

The final multivariable linear mixed-effects model comparing change over time in the cJADAS in the pre-intervention and Phase I cohorts included the following covariates: baseline cJADAS, time (in months), pain VAS, tender entheses count, biologic use and an interaction between incident diagnosis and time (Table 2).

**Table 2:** Estimates of factors associated with change in cJADAS over time in mixed-effects models in pre-intervention and Phase I implementation cohorts

	Univariate*		Multivariate	
	β (95% CI)	***p***-value	β (95% CI)	***p***-value
Male Sex	−1.86 (−2.75,-0.97)	< 0.01	–	–
Non-white race	−1.17 (− 1.42,1.08)	0.08	–	–
Public insurance	0.78 (− 0.08,1.65)	0.08	–	–
Baseline cJADAS	0.48 (0.42,0.55)	< 0.01	0.34 (0.28,0.40)	< 0.01
Polyarticular disease	1.47 (0.52,2.42)	< 0.01	–	–
Incident diagnosis	2.53 (1.60,3.47)	< 0.01	0.83 (0.02,1.63)	0.04
Time (in months)	−0.11 (− 0.16,-0.05)	< 0.01	− 0.03 (− 0.07,0.00)	0.07
Incident diagnosis * time interaction^	− 0.24 (− 0.35,-0.11)	< 0.01	− 0.21 (− 0.29,-0.13)	< 0.01
Pain VAS ^#^	0.99 (0.88,1.10)	< 0.01	0.76 (0.66,0.86)	< 0.01
Tender entheses count ^#^	0.66 (0.51,-0.80)	< 0.01	0.14 (0.02,0.25)	0.02
Biologic use ^#^	−1.48 (−2.21,-0.75)	< 0.01	−0.56 (− 1.06,-0.07)	0.02
DMARD use ^#^	0.46 (−0.43,1.35)	0.31	–	–
Phase I cohort (vs pre-intervention)	0.73 (−0.08-,1.54)	0.66	–	–
Pre/post Phase I cohort * time interaction^	0.02 (−0.21,0.24)	0.16	–	–

* Marginal effect of each covariate on the repeated measured JADAS over time. # Repeated measures collected at every visit. Interaction terms represent whether there is a significant difference in slope/rate of change over time in those with * recent versus prevalent diagnosis and ^before and after the Phase I implementation

*Abbreviations*. *cJADAS* clinical Juvenile Arthritis Disease Activity Score, *CDS* clinical decision support tool, *VAS* visual analogue scale, *DMARD* Disease Modifying Anti Rheumatic Drug

Each 0.7 point increase in pain was independently associated with a 1.0 point higher cJADAS at any time point (*p* < 0.01). Higher baseline disease activity and number of tender entheses at each visit were also significantly associated with higher cJADAS at each timepoint (Table 2). There was no significant interaction between tender entheses and pain (*p* = 0.77 for interaction). Incident cases had a greater rate of improvement in cJADAS over time (predicted mean change − 2.95 at 12 months, 95% CI [− 2.12, − 3.78], *p* < 0.01) compared to prevalent cases (− 0.41, 95% CI [− 0.84, 0.03], *p* = 0.07). There was no significant difference in the cJADAS over time in children in the pre-intervention and Phase I cohorts (β = 0.73 95%CI: − 0.08, 1.54; *p* = 0.08). There was also no significant difference in slope/rate of change over time in the pre-intervention and Phase I cohorts (interaction of cohort and time β = 0.02 95%CI: − 0.21, 0.24; *p* = 0.16).

Of the cJADAS components, the provider global assessment and joint count improved over time among incident cases (predicted mean change at 12 months − 1.06, 95% CI [− 1.41, − 0.71], *p* < 0.01 and − 1.70, 95% [− 2.30, − 1.10], p < 0.01, respectively). However, there was no improvement over time in the patient global assessment for either incident or prevalent cases.

### Impact of treat-to-target intervention on patient-reported pain

Table 3 shows the results of univariate and multivariable mixed-effects models evaluating change in pain over time in the pre-intervention and Phase I cohorts. The final multivariable model comparing change over time in patient-reported pain consisted of the following covariates: male sex, baseline pain score, incident (versus prevalent) diagnosis, and the cJADAS.

**Table 3:** Estimates of factors associated with pain over time in mixed-effects models in pre-intervention and Phase I implementation cohorts

	Univariate*		Multivariate	
	β (95% CI)	***p***-value	β (95% CI)	***p***-value
Male Sex	−1.74 (− 2.22,-1.26)	< 0.01	− 0.49 (− 0.83,-0.15)	< 0.01
Public insurance	0.37 (− 0.14,0.87)	0.16	–	–
Baseline pain	0.58 (0.51,0.65)	< 0.01	0.38 (0.31,0.45)	< 0.01
Incident diagnosis*	0.91 (0.26,1.37)	< 0.01	− 0.59 (− 0.94,-0.24)	< 0.01
cJADAS ^#^	0.39 (0.35,0.43)	< 0.01	0.31 (0.27,0.35)	< 0.01
Time (in months)	−0.02 (− 0.05, 0.01)	0.28	0.00 (− 0.02, 0.02)	0.65
Tender entheses count ^#^	0.38 (0.29,0.48)	< 0.01	–	–
DMARD use ^#^	0.09 (− 0.45,0.63)	0.73	–	–
Biologic use	− 0.37 (− 0.83,0.09)	0.12	–	–
Pre-intervention vs Phase I cohort	0.49 (− 0.09,1.07)	0.10	–	–
Pre/post Phase I cohort * time interaction^	−0.03 (− 0.17,0.11) p	0.67	–	–

* Marginal effect of each covariate on the repeated measured JADAS over time. # Repeated measures collected at every visit. Interaction term represents whether there is a significant difference in slope/rate of change over time in those before and after the polyarticular CDS intervention

*Abbreviations*. *cJADAS* clinical Juvenile Arthritis Disease Activity Score, *CDS* clinical decision support tool, *VAS* visual analogue scale, *DMARD* Disease Modifying Anti Rheumatic Drug

There was no significant change in pain over time (*p* = 0.65). Higher pain score at cohort entry was significantly associated with higher magnitude of pain at any timepoint (*p* < 0.01). In contrast to disease activity, children with an incident diagnosis did not have a greater rate of improvement in pain over time compared to prevalent cases (*p* = 0.20 for interaction). Male sex was also significantly associated with lower pain scores (p < 0.01). There was no significant difference in the magnitude of pain over time (β = 0.49 95%CI: − 0.09,1.07; *p* = 0.10) or the rate of change in pain in children in the pre-intervention versus Phase I cohorts (interaction of cohort and time β = − 0.03 95%CI: − 0.17,0.11; *p* = 0.67).

### Impact of ERA-clinical decision support on disease activity

Table 4 shows the results of univariate and multivariable mixed-effects models evaluating change in cJADAS over time in the pre-intervention and Phase II cohorts. In the Phase II cohort, 51 patients had ≥2 visits, 41% of whom were an incident diagnosis. For these patients, treating providers completed a target attestation in 88% of visits. The final multivariable model comparing change over time in the cJADAS between the pre-intervention and Phase II cohorts consisted of the following covariates: baseline cJADAS, incident (versus prevalent) diagnosis, and pain VAS.

**Table 4:** Estimates of factors associated with cJADAS over time in mixed-effects models in pre-intervention and Phase II implementation cohorts

	Univariate *		Multivariate	
	β (95% CI)	***p***-value	β (95% CI)	***p***-value
Age	0.61 (−0.32,-0.00)	0.05	–	–
Male Sex	−1.62 (− 2.77,-0.46)	< 0.01	–	–
Public insurance	−0.38 (− 1.50,0.74)	0.50	–	–
Baseline cJADAS	0.61 (0.52,0.69)	< 0.01	0.47 (0.38,0.55)	< 0.01
Polyarticular disease	0.80 (−0.40,1.99)	0.19	–	–
Sacroiliitis (ever)	−1.32 (− 2.57,-0.08)	0.04	–	–
Incident diagnosis	3.12 (2.00,4.20)	< 0.01	0.26 (−0.52,1.03)	0.52
Time (in months)	−0.17 (− 0.33,− 0.01)	0.04	-0.01 (− 0.13, 0.10)	0.81
Incident diagnosis* time interaction^	− 0.39 (− 0.75,-0.02)	0.04	−0.32 (− 0.56,-0.08)	< 0.01
Pain VAS ^#^	0.94 (0.79,1.09)	< 0.01	0.72 (0.60,0.85)	< 0.01
Tender entheses count ^#^	0.57 (0.40,0.74)	< 0.01	–	–
Biologic use ^#^	−2.02 (− 3.02,-1.01)	< 0.01	–	–
Pre-intervention vs Phase II cohort	0.33 (−0.77,1.44)	0.56	–	–
Pre-intervention vs Phase II cohort * time interaction^	−0.04 (− 0.36,0.29)	0.82	–	–

Legend**.** ^**a**^Pain visual analog scale score (range 0,10); ^b^cJADAS = three-variable clinical Juvenile Arthritis Disease Activity Score (range 0,30); ^c^Active joint count (range 0,10); ^d^Physician global assessment of disease activity visual analog scale (range 0,10); ^e^Patient/parent global assessment of disease activity visual analog scale (range 0,10); ^f^ (range 0,14) ^g^B27 status missing for 4 subjects

*Abbreviations*: *CDS* Clinical Decision Support, *IQR* interquartile range, *ILAR* International League of Associations for Rheumatology, *ERA* Enthesitis Related Arthritis, *SIJ* Sacroiliac Joints, *HLA* Human Leukocyte Antigen, *MRI* Magnetic Resonance Imaging, *DMARD* Disease modifying Anti Rheumatic Drug

Higher cJADAS at cohort entry (*p* < 0.01) and higher pain at each visit (p < 0.01) were significantly associated with higher cJADAS over time. There was no significant difference in the cJADAS over time in children in the pre-intervention versus Phase II cohorts (β = 0.33 95%CI: − 0.77, 1.44; *p* = 0.56). There was also no significant difference in rate of cJADAS change over time in the pre-intervention versus Phase II cohorts (β = − 0.04 95%CI: − 0.36, 0.29; *p* = 0.82).

### Sensitivity analysis for target attestation

In the sensitivity analysis of the Phase II cohort which included only patients for whom target attestation was completed at all visits by the treating physician (42 patients, 31% incident cases), higher disease activity at cohort entry (*p* < 0.01) and pain at each visit (p < 0.01) were the only variables significantly associated with change in cJADAS over time. There was no significant difference in the cJADAS over time or in the rate of cJADAS change in children in the pre-intervention versus Phase II cohorts.

## Discussion

This is the first study to evaluate if standardized point-of-care disease activity monitoring with CDS, as compared to standard care, improves disease activity and patient-reported pain in children with ERA. Although we did not observe a significant impact of the interventions on disease activity scores or pain, there are several important findings from our study that have implications for treat-to-target in children with ERA. First, several pathognomonic disease features of ERA were not specifically captured by the cJADAS score and may account for the lack of impact of the intervention on children with ERA despite previously reported improvements among children with the polyarticular subtypes of JIA. Second, pain and tender entheses count were significant determinants of disease activity scores over time and should be assessed as part of standard of care. Lastly, regardless of implementation phase, incident cases had a significantly greater rate of improvement in cJADAS scores over time compared to prevalent cases, but not in pain, which highlights the importance of targeting pain in this population.

There are several reasons we may not have detected a significant clinical impact of the intervention on children with ERA, despite having previously demonstrated its effectiveness for polyarticular JIA. Although the cJADAS is validated all non-systemic subtypes of JIA and widely used in clinical studies, including clinical trials, it may not be the ideal outcome metric for treat-to-target in ERA. First, cJADAS scores are heavily weighted on peripheral joint count. The majority of ERA patients had oligoarticular disease with low cJADAS scores at baseline, so unlike children with polyarticular RF+ and RF- subtypes of JIA, there was not a lot of room to detect improvement. Second, the Phase I CDS algorithms were designed to improve cJADAS in children with a polyarticular disease course, therefore efforts to standardize changes in systemic therapy targeted toward reductions in joint count were less likely to benefit ERA patients. Lastly, there are many aspects of disease activity specific to ERA that are not included in the cJADAS, such as axial symptoms, entheseal tenderness and pain. For many children with SpA, peripheral arthritis is only one of many disease features that need to be addressed. While other clinical disease features may be captured in the physician disease activity score or the patient/caregiver global assessment components of the cJADAS, they could also be overlooked. Furthermore, the importance or “weighting” of the various disease features in the overall physician assessment likely varies widely amongst physicians. Using a composite disease activity score like the juvenile spondyloarthritis disease activity (JSpADA) index might solve this issue, as peripheral joint count is only 1 of 8 different disease activity metrics [18]. Our study highlights the need to both standardize assessment of other disease features unique to ERA, and define disease activity states for tools such as the JSpADA index so that appropriate outcome metrics can be used in future treat-to-target endeavors.

Tender entheses was one particular disease feature that was associated with disease activity over time. It was not, however, a significant predictor of higher pain over time after adjusting for baseline pain, and the effect of pain on disease activity scores was not modified by tender entheses count. Refractory enthesitis often requires escalation of systemic therapy to tumor necrosis factor inhibitors or other biologic agents. While inactive arthritis is a treatment target in most children, the ideal treatment target with respect to enthesitis in children is not entirely clear. The ERA-specific CDS algorithms in our study were developed to address arthritis and sacroiliitis only, and not peripheral enthesitis. As such, changes in systemic therapy to target enthesitis remained unstandardized. Efforts to achieve greater consensus on the treatment target for enthesitis and which patients with refractory enthesitis warrant more aggressive therapy are needed before treat-to-target can be fully implemented for ERA. Future iterations of CDS for ERA will also need to include specific algorithms to address enthesitis.

Another important finding from our study was that pain remained unchanged over time, regardless of implementation period. Even among incident ERA cases, pain persisted over time despite improvements in cJADAS. Furthermore, baseline pain was a strong predictor of higher pain over time in our cohort, and adjusting for baseline pain completely ameliorated any differences in pain pre and post-implementation. This is consistent with prior studies of JIA, in which self-reported pain at baseline was also associated with greater pain at long-term follow-up, in addition to worse functional outcomes and lower rates of remission [4, 5, 19]. However, in contrast to the large Nordic JIA cohort, of which only a small subset of patients had ERA, average pain intensity in our cohort of children with ERA did not decrease. Pain was an important determinant of disease activity, independent of tender entheses count, and may explain why improvements in physician global assessments and joint count were not paralleled by similar improvements in patient global assessment scores. Current treat-to-target paradigms do not specifically address pain, which may explain the limited impact of our intervention on children with ERA despite its effectiveness for children with polyarticular JIA. The new Outcome Measures in Rheumatology (OMERACT) core domain set for JIA now includes pain as a mandatory domain, which was prioritized by parents/patients [20]. Pain can either be an indication of ongoing inflammation requiring more aggressive therapy, or that another non-pharmacologic strategy is needed to treat residual symptoms. As children with the ERA subtype of JIA are more likely to have persistent pain, future iterations of treat-to-target for ERA in particular need to both systematically assess and treat pain, as well as identify when pain represents a need for escalation of systemic treatment versus adjunct therapies to address residual symptoms.

## Conclusions

In summary, we report results of standardized point-of-care disease activity monitoring and CDS intervention in patients with ERA at a single tertiary care center. Our results did not show a significant impact of the intervention, but they do reflect successful implementation of standardized point-of-care disease activity monitoring and CDS interventions in routine clinical care. Our negative results highlight several important considerations for future studies of standardized disease activity monitoring and CDS in ERA, including the need to account for heterogeneity of ERA disease features, use of alternative disease activity metrics to better capture improvements in clinical outcomes relevant to juvenile spondyloarthritis, and the need for better strategies to systematically assess and address patient-reported pain.

## Data Availability

The datasets used and/or analysed during the current study are available from the corresponding author on reasonable request.

## Abbreviations

CDS: Clinical Decision Support
CHOP: Children’s Hospital of Philadelphia
cJADAS: Clinical Juvenile Idiopathic Arthritis Disease Activity Score
DMARD: Disesase Modifying Anti Rheumati Drug
EHR: Electronic Health Record
ERA: Enthesitis Related Arthritis
HLA: Human Leukocyte Antigen
IQR: Interquartile Range
ILAR: International League Against Rheumatism
JIA: Juvenile Idiopathic Arthritis
MRI: Magnetic resonance Imaging
PROMIS: Patient-Reported Outcomes Measurement Information System
PRO: Patient Reported Outcome
REDCap: Research Electronic Data Capture
TTT: Treat-to-target
VAS: Visual Analog Scale
